# Reflection and self‐efficacy for clinical skills

**DOI:** 10.1111/tct.13833

**Published:** 2024-11-11

**Authors:** Jade Lene Yong, Gwyndaf Roberts

**Affiliations:** ^1^ Newcastle University Medicine Malaysia (NUMed) Johor Malaysia; ^2^ School of Medicine, Faculty of Medical Sciences Newcastle University Newcastle Upon Tyne UK

## Abstract

**Introduction:**

Clinical skills are fundamental to medical school curriculums and typically introduced within the preclinical years. In their experiential learning, students' self‐efficacy, or the belief in their ability to succeed, is an important factor in influencing clinical skill mastery. Reflection is thought to affect self‐efficacy; however, its exact impacts remain largely unexplored within published literature. This mixed methods study investigated whether preclinical students' engagement with reflection affected self‐efficacy for clinical skills.

**Methods:**

Two hundred seventy‐three of the 289 preclinical medical students who were invited to participate responded to this 2022 study. We used validated questionnaires to measure engagement with reflection and perceived self‐efficacy for clinical skills, conducting hierarchical multiple linear regression for analysis. Thirteen students participated in semi‐structured interviews and focus groups, which were analysed via thematic analysis.

**Results:**

While statistical analysis showed no significant effects of engaging with reflection on clinical skill self‐efficacy, thematic analysis suggested that students perceived the opposite. The themes through which reflection affected self‐efficacy were by ‘evaluation of performances’ against expected outcomes, ‘familiarisation and understanding of skills’, by ‘transforming personal mindsets’ and allowing students to ‘connect to their emotions’.

**Conclusion:**

This study suggests that engaging with reflection can positively or negatively affect self‐efficacy for clinical skills, depending on students' attitudes towards reflective practice. Solely engaging with reflection is insufficient to alter self‐efficacy beliefs and should be considered alongside personal factors including the individual's mindset and perceived need for reflection. The medical educator's role in facilitating reflection is important, enabling students to reap the benefits of this practice.

## 1 INTRODUCTION

Clinical skills, a term encompassing a range of abilities such as history taking, physical examination and undertaking technical procedures, form the foundations of a doctor's practical skillset.^1^ Although there is a strong psychomotor element to most clinical skills, resulting in a belief that they are easily achievable through repeated practice,^2^ their successful acquisition is more complex than this. To develop these skills, students are often immersed into an experiential learning cycle,^3^ where they carry out a skill, reflect on this experience and conceptualise their thoughts into action plans for improvement. These new ideas are then incorporated into their next attempt with the skill. Studies have shown that engaging in this cycle improves learners' clinical skill performance, confidence levels and the effectiveness of their learning.^4^, ^5^ Reflection is thought to be a key driver in the improvement of these skills, alongside deliberate practice and critical feedback.^6^

This practice of reflection can be related to Bandura's theory of self‐efficacy, which refers to one's beliefs about their own capabilities to achieve their goals^7^; students with a stronger sense of self‐efficacy are thought to be motivated, high‐achieving individuals and likely to make better learning decisions.^8^ In the context of clinical skills, although knowledge and repetition may be sufficient to execute a skill well, it is arguably the student's self‐efficacy, or beliefs in their potential to accomplish the skill that has a major impact on its mastery.^9^ Bandura^10^ suggested that an individual's ability to reflect may alter their thinking and could potentially affect self‐efficacy; however, the precise effects of reflection on self‐efficacy are still largely unexplored in published literature, let alone that of self‐efficacy specifically for clinical skills.

We conducted our study within the preclinical medical student population (Years 1 and 2, henceforth referred to as ‘preclinical students’) as clinical skills are typically introduced at this point in one's medical education,^11^ and their early acquisition is thought to ease transitions into the more practical phase of medical school (Year 3 onwards).^12^ Understanding this topic could contribute to better clinical skill learning experiences for these students.

Our study aimed to examine the impact of reflection on preclinical students' self‐efficacy for clinical skills. While the impacts of deliberate practice and feedback on skills development have been investigated,^13^ the role of reflection is perhaps less well‐understood. To answer our research question: ‘What is the effect of preclinical students' engagement with reflection on their self‐efficacy for clinical skills?’, we took a post‐positivist approach^14^ and defined ‘engagement with reflection’ as how frequently the students examined their thoughts, feelings and actions.

## 2 METHODS

This research was conducted in 2022, with the study population being the 2021–22 cohort of Newcastle University Medicine Malaysia (NUMed) preclinical students (*n* = 289). These students were taught the principles of reflection through an induction lecture and seminar in their first year. These sessions introduced students to the three‐stage model of reflection^15^ and provided practical examples of how and when they could undertake this practice. Students also engaged with case‐based reflective exercises to gain exposure and understanding on incorporating reflection into their studies. Reflective skills were further developed during clinical skills sessions, where tutors facilitate the process each time a student newly performs a skill. Based on Kolb's experiential learning cycle,^3^ they would encourage students to reflect on the encounter, what they had done well and could improve on. The curriculum further incorporates reflection on clinical skills by the use of a reflective portfolio which students populate whenever they attempt a new skill. Thus, despite inherent differences between individual students, the study population would have comparable understandings of the reflective cycle and its role in improving clinical skills.

For our mixed methods study, we opted for a convergent design.^16^ Collecting quantitative and qualitative data in parallel, we analysed both datasets separately before combining them for an integrated evaluation. This allowed for triangulation of both data sources to test our hypothesis: That preclinical students' engagement with reflection affects their self‐efficacy for clinical skills. To ensure rigour, we referred to published criteria for mixed methods studies^17^, ^18^ to ensure transparency in outlining our research process.

All 289 NUMed preclinical students were offered the opportunity to fill in physical questionnaires during their compulsory clinical skills sessions (convenience sampling). Interest forms were included with the study questionnaires for students to volunteer their participation for the interviews and focus groups, and invitations were sent out to the whole preclinical student cohort via email and social media messaging applications. A total of 273 students responded to the quantitative data collection, with 13 participating in the qualitative component.

### 2.1 Quantitative methods

To measure ‘engagement with reflection’ and ‘self‐efficacy for clinical skills’, we utilised the Self‐Reflection and Insight Scale (SRIS)^19^ and the Learning Self‐Efficacy Scale (L‐SES) for Clinical Skills,^20^ respectively. Both were 5‐point Likert scales validated for use amongst undergraduate medical students and deemed reliable (Cronbach's alpha >0.8).^19^, ^20^ The finalised study questionnaire also included a demographics section (participant age, gender, year group).

The data collected were analysed using IBM SPSS Statistics software (Version 27.0)^21^ and treated as parametric, replicating methods used by the scales' developers, supported by statistical reasoning that the computed data would overwrite the ordinal characteristics of a single Likert item.^22^

Our study specifically examined the effects of the ‘Engagement with Self‐Reflection’ subscale of SRIS on ‘Total Self‐Efficacy’, computed from the sum of cognitive, affective and psychomotor domains in L‐SES. To do this, we used hierarchical multiple linear regression to control for covariates and potential variables of interest, which potentially had effects on self‐efficacy, while ensuring that our data met the assumptions required for this statistical analysis.^23^

The hierarchical multiple regression was conducted in three stages: Firstly, Year Group, Gender and Age covariates were entered at Stage 1 of the regression model to control for demographics. The Need for Self‐Reflection and Insight variables were entered at Stage 2. This ensured that the independent variable, Engagement with Self‐Reflection, could be analysed in isolation when entered into Stage 3 of the model. The null hypothesis was ‘There is no effect of preclinical students’ engagement with reflection on their self‐efficacy for clinical skills'. A *p*‐value of <0.05 was considered significant, with confidence intervals set at 95%.

An Analysis of Variance (ANOVA) was also conducted to confirm whether our regression model could predict the dependent variable.

### 2.2 Qualitative methods

We conducted four individual semi‐structured interviews with Year 2 students and two focus groups involving nine Year 1 students in total (groups of four and five), accommodating participant availability. As Year 1 students were less familiar with the process of reflection, the use of focus groups was preferable due to their dynamic and synergetic nature, allowing more opinions to be discussed. Conversely, individual interviews with Year 2 students provided the opportunity to explore their perspectives further. Each session lasted an hour and was conducted online via Zoom. Referring to the origins of self‐efficacy proposed by Bandura^7^ and Maddux,^24^ we ensured that we explored participants' perceptions on the role of reflection in affecting each of its sources positively and negatively and how this ultimately impacted their self‐efficacy for clinical skills. As a form of triangulation, we asked participants about the effects of reflection on their self‐efficacy in terms of cognitive, affective and psychomotor domains as categorised in the L‐SES questionnaire. The semi‐structured nature of our methods allowed us to evolve our interview schedule (Box 1), improving the clarity and quality of questions throughout the process.

BOX 1 **Key questions of interview schedule.**

Key questions:

1 How does [*insert point from Forms of Reflection in table below*] affect your clinical skills performance and, ultimately, your self‐efficacy for them?

**Table:** 

**Sources of self‐efficacy**	**Forms of reflection**
Performance accomplishments	Reflection on actual clinical skills experiences
Vicarious experiences	Observation of peers carrying out the skills (often involves providing feedback on their performance)—serves as a reflection on one's own performance of the skill.
Verbal persuasion	Reflection on feedback given by facilitators and peers on one's performance, as well as the self‐talk that an individual may engage in with regard to their clinical skills
Emotional arousal	Being mindful and aware about one's emotions in relation to their clinical skills experiences
Imagined experiences	Reflection in the form of mental visualisation of oneself performing the skills without physically executing the motions

2 What are the benefits or drawbacks of reflection on learning clinical skills? *(Probe for effects on Understanding, Interest/Enjoyment, Performance)*

Interview recordings were transcribed verbatim and analysed with Braun and Clarke's^25^ proposed steps of thematic analysis. The coding process was undertaken by generating codes directly from transcripts through a predominantly deductive approach, using the research question to guide the analysis in identifying the effects of reflection on self‐efficacy for clinical skills, with some element of inductivity, allowing the data to guide the analysis for a richer variety of codes. A mixture of semantic and latent codes was developed and re‐evaluated throughout.

Once the whole dataset was coded, preliminary themes were reviewed by the lead researcher, checking that they were well‐defined and supported by coherent, meaningful data. The interpretation and identification of the themes were discussed and agreed on by the authors to ensure rigour. The final themes were chosen and named, ensuring that they captured the essence of the coded data within them.

### 2.3 Ethics statement

Approval for the study was obtained from the Newcastle University Faculty of Medical Sciences Research Ethics Committee.

## 3 RESULTS

3.1

From our sample of 289 NUMed preclinical students, 273 (94.5%) responded to the study questionnaire. Of these, 173 (63.4%) were females, and 146 (53.5%) were second‐year students. The mean participant age was 20 years.

### 3.2 The relationship between engagement with reflection and self‐efficacy for clinical skills

Table 1 presents the correlation matrix of the variables involved in our analysis. The independent variable was ‘Engagement with Self‐Reflection’ and the dependent variable was ‘Total Self‐Efficacy’. ‘Year Group’, ‘Gender’ and ‘Age’ were demographic covariates (entered into Stage 1 of the regression model), while ‘Need for Self‐Reflection’ and ‘Insight’ were potential variables of interest (Stage 2). The correlation between Engagement with Self‐Reflection and Total Self‐Efficacy is positive (0.252). Total Self‐Efficacy was also significantly positively correlated with Need for Self‐Reflection and Insight (0.302 and 0.343, respectively).

**TABLE 1:** Correlation matrix and descriptive statistics for Total Self‐Efficacy, demographics, need for self‐reflection, insight and engagement with self‐reflection.

	Variable	1	2	3	4	5	6	7
1	Total Self‐Efficacy	1.000						
2	Year Group	−0.042	1.000					
3	Gender	0.010	0.061	1.000				
4	Age	−0.045	0.322*	0.035	1.000			
5	Need for Self‐Reflection	0.302*	0.033	0.075	0.048	1.000		
6	Insight	0.343*	−0.037	−0.167*	−0.013	0.163*	1.000	
7	Engagement with Self‐Reflection	0.252*	0.045	0.081	0.080	0.732*	0.250	1.000

*Note*: *N* = 261 (12 cases excluded listwise from original dataset due to missing data).

* are statistically significant correlation coefficients (p<0.05).

From the ANOVA test, the F‐statistic for the model at Stage 3 was 9.626, *p* < 0.001, suggesting that the final model improved our overall ability to predict Total Self‐Efficacy.

Table 2 summarises the results of the three‐stage hierarchical multiple regression. The ‘Need for Self‐Reflection’ and ‘Insight’ variables were introduced into the model at Stage 2 and significantly accounted for an additional 18.2% of variance in the outcome, with an *R* ^*2*^ change of 0.182, *F*(2, 255) = 28.494, *p* < 0.001. Hence, they significantly improved the ability of the model to predict Total Self‐Efficacy.

**TABLE 2:** Results of hierarchical multiple regression analysis for variables predicting Total Self‐Efficacy and model summaries for each stage (final column).

	Unstandardised coefficients		Standardised coefficients			95.0% confidence interval for B		*R*	*R* Square	*R* Square change	*F* change	*p*
Variable	*B*	Std. error	Beta	*t*	*p*	Lower bound	Upper bound					
Stage 1								0.055	0.003	0.003	0.260	0.85
Year Group	−0.353	0.737	−0.032	−0.478	0.63	−1.805	1.099					
Gender	0.147	0.725	0.013	0.203	0.84	−1.281	1.576					
Age	−0.150	0.279	−0.035	−0.535	0.59	−0.700	0.401					
Stage 2								0.430	0.185	0.182	28.494	<0.001
Year Group	−0.300	0.670	−0.027	−0.448	0.65	−1.619	1.018					
Gender	0.527	0.671	0.045	0.785	0.43	−0.795	1.848					
Age	−0.195	0.254	−0.046	−0.768	0.44	−0.695	0.305					
Need for Self‐Reflection	0.345	0.079	0.252	4.361	<0.001	0.189	0.501					
Insight	0.396	0.075	0.308	5.278	<0.001	0.248	0.544					
Stage 3								0.430	0.185	0.000	0.040	0.84
Year Group	−0.298	0.671	−0.027	−0.444	0.66	−1.620	1.023					
Gender	0.537	0.674	0.046	0.796	0.43	−0.791	1.864					
Age	−0.192	0.255	−0.045	−0.753	0.45	−0.694	0.310					
Need for Self‐Reflection	0.362	0.114	0.264	3.168	<0.05	0.137	0.586					
Insight	0.399	0.077	0.310	5.195	<0.001	0.248	0.551					
Engagement with Self‐Reflection	−0.023	0.114	−0.017	−0.201	0.84	−0.247	0.201					

However, when independent variable Engagement with Reflection was added to Stage 3, it only accounted for <0.01% of variance in Total Self‐Efficacy, failing to make a significant contribution to the final model. Since the *p*‐value for this stage is >0.05, the null hypothesis was not rejected, suggesting that there is no significant effect of preclinical students' engagement with reflection on their self‐efficacy for clinical skills.

### 3.3 Impacts of engaging with reflection on self‐efficacy for clinical skills

All 13 interview and focus group participants experienced engaging with reflection during their studies in relation to Bandura's and Maddux's sources of self‐efficacy (Box 1). Students reported reflecting on their clinical skills experiences in the form of internal mental processes, written reflections and verbally in teaching sessions or discussions.

**FIGURE 1:**
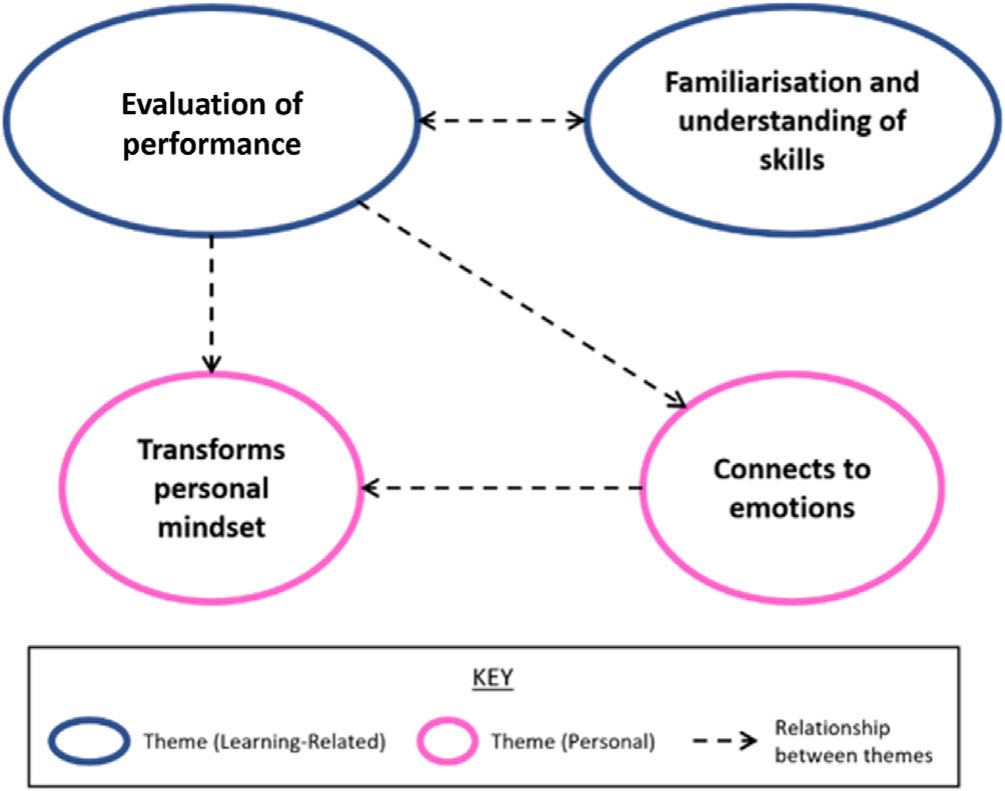
Thematic map of the impacts of preclinical students' engagement with reflection on self‐efficacy for clinical skills.

Four key themes on how engagement with reflection impacted self‐efficacy for clinical skills were identified: ‘evaluation of performance’; ‘familiarisation and understanding of skills’; ‘transforms personal mindset’; and ‘connects to emotions’. The first two were closely related to the learning experience, while the other two were more associated with the individuals themselves. The relationships between the themes depicted by the arrows in Figure 1 are explained within the text.

#### 3.3.1 Theme: Evaluation of performance

By reflecting on clinical skill experiences and distancing themselves from previous encounters, students felt like they could appraise their performances. Reflection was described as a process that enabled participants to identify and analyse personal weaknesses, allowing them to generate concrete solutions to overcome them.

Participant 1 (P1) ‘The more you reflect, the more you understand where you went wrong. When you start doing that, clinical skills come to you more easily. And maybe that would help you build your [self‐efficacy]’;

P2 ‘When I reflect, like when I write it down and look back, I do think, “Oh, I did not know that I struggled with that”’

This is important because it allows students to gauge their competency levels against expected outcomes, indirectly helping to inform their self‐efficacy for clinical skills.

However, casting too critical of an eye over one's performance could also backfire, causing distress:

P3 ‘I overthink a lot, so I have a lot of moments where if I reflect too much, I start self‐criticising, nit‐picking on the things that I did [during the class]’.

P4 ‘Sometimes, depending on how I perceive it, reflection just brings me down, and I ask myself “Why did I not do that?” or “Have I done that?”’

As opposed to a conducive reflection, rumination on a particular learning experience could be detrimental. In overthinking prior performances and scrutinising actions, which may not have been an actual issue, one's self‐efficacy could be significantly undermined.

#### 3.3.2 Theme: Familiarisation and understanding of skills

This theme refers to one's general level of comfort with their knowledge, understanding and exposure to clinical skills. Participants agreed that there was a mutual relationship between this and the previous theme, whereby going through multiple **evaluations of one's performance** encouraged repetition and familiarity, enabling them to assess their performances more accurately.

For many participants, reflection served as a reminder and reinforcement of the steps they should take to improve their skills. Most of the time, revisiting their learning experiences piqued curiosity about the skill, encouraging students to deepen their clinical skill understanding in order to enhance performance. Hence, by engaging with the cognitive aspects of a skill, reflection could positively impact one's self‐efficacy:

P1: ‘When we reflect a lot more on clinical skills, we can understand the concepts. You will get the assurance that you are doing something right, and when you do it properly, I think you [build self‐efficacy]’.

Furthermore, increased familiarity and understanding of a skill invoked self‐efficacy when students felt competent enough to point out another's mistake:

P5: ‘I think that reflecting also adds to my self‐efficacy when I give feedback [to others], because in the moment I pick up their mistakes, I feel like I did enough preparation for [the skill]’.

However, this could lead to an opposite reaction, with the self‐imposed expectation of perfection in their own performances:

P6: ‘[Being able to point out someone's mistake] personally deflates my [self‐efficacy], because now I'm constantly thinking at the back of my mind – the number of steps I have to do, the procedure and more’.

Comparing the two perspectives, it is interesting to note how differently reflection could impact self‐efficacy, albeit through similarly reflecting on others' performances. With the latter participant feeling more pressured after this experience, there is perhaps an element of one's personal mindset that also contributes to self‐efficacy beliefs.

#### 3.3.3 Theme: Transforms personal mindset

Through reflection, students also reported changes to how they perceived themselves, in terms of their outlook and awareness of their personal progress and capabilities.

Since reflection was often carried out after a clinical skill was attempted, many students regarded the process as a milestone or symbol of the skill's completion. This resulted in a sense of achievement, which cemented positive self‐efficacy beliefs in being able to repeat their performance, providing students' reassurance with their previous successful attempts:

P2 ‘Actually, reflecting on a skill is almost like achieving that skill. It's like “Oh, I've done [the skill] and I know it, so [it] can be done again”’;

P7 ‘Reflection gives me a bit more confidence moving forward. [If] I did something well before, chances are I can do it well next time’.

However, as suggested earlier, reflection on mistakes sometimes caused self‐doubt, which adversely affects self‐efficacy for clinical skills. Participants suggested that students tend to blame themselves for bad performances, labelling themselves as ‘incompetent’ or ‘forgetful’ despite external factors such as time, experience and the environment being important factors in improving one's skills:

P7 ‘The thing is, it's easy to fall into the habit of thinking “If I do something wrong, then it's something wrong with me” … We try to connect [our mistakes] back to ourselves. If we forget a step, we think we are forgetful. But the thing is, it's not that. It just happened that your brain just short‐circuited at that moment’.

P8 ‘Sometimes, I pin my actions to myself; if I do something wrong, I think it's a problem with me. So sometimes, reflection lowers my [self‐efficacy]’.

With this negative mentality, students experienced a decrease in their self‐efficacy, finding it harder to believe in their abilities to execute the skills. However, consistent engagement with reflection may enable one to **evaluate their performances** to monitor their progress and improvement over time, greatly improving their personal view of their skillset. In this sense, reflection could build self‐efficacy through realisation that a previously challenging task has now become second nature and could also encourage realistic expectations of an individual's level of competence:

P2 ‘Reflection allows you to observe the transition between your challenges. They help me see that at one point, I saw [a clinical skill] as a challenge, but now it has become almost something I do very effortlessly’.

P3 ‘With reflection, I realised it's okay to have so many things to improve on. We're still learning’.

The latter quote implies that reflection may help students to realise that they are still in the process of learning. Participants agreed that effective reflections increased self‐awareness of personal capacities, encouraged them to be kinder to themselves and thus promoted self‐efficacy beliefs.

#### 3.3.4 Theme: Connects with emotions

Lastly, participants perceived that reflection allowed them to connect more deeply with their emotions, which impacted their self‐efficacy.

For many students, reflection on past mistakes sometimes uncovered feelings of inadequacy, which spiralled into emotions ranging from anxiety, frustration, sadness, guilt to fear. These negative emotions would severely impact on one's self‐efficacy:

P3 ‘Reflection does really cause me a lot of stress sometimes … But more than anxiety, is just … depressed feelings … A bit of imposter syndrome, I guess’.

P5 ‘Personally, overthinking a mistake when reflecting sometimes makes me more anxious. Sometimes, just from a clinical skill mistake, it extends to a whole crisis, where I think “Oh, am I really suited for [medical school]?”’

By tapping into one's feelings on a negative experience, it appears that reflection could potentially trigger a chain of strongly pessimistic reactions. This could escalate into participants questioning if they deserved to be medical students or even suited to the profession. These thoughts are destructive towards one's self‐efficacy and, if sustained, may **transform one's mindset** into one of self‐doubt and insecurity, further impairing self‐efficacy beliefs.

Nevertheless, participants felt that there were still benefits to connecting with their emotions, as reflection enabled them to **evaluate previous performances** and look forward to reattempting the skill:

P9 ‘After reflecting, I'm ready to try [the clinical skill] again, so I just want to do it … I'm just excited to do it again because I want to correct where I went wrong. I want to do a perfect run, so I'll feel enthusiastic for the second run’.

P3 ‘I look forward to practising that clinical skill again [after reflecting]. Because when you reflect, you have identified ways you can improve, and I think there's that urge to want to automatically do the better thing now, with your improved [self‐efficacy]’.

Building on this, students felt that these positive emotions reinforced their self‐efficacy beliefs that they would be able to excel and improve on their clinical skills in the future.

## 4 DISCUSSION

This study sought to examine the effects of preclinical students' engagement with reflection on their self‐efficacy for clinical skills. The results from both datasets appear diverged; the quantitative data suggest that there are no statistically significant effects of engagement with reflection on one's self‐efficacy for clinical skills, yet the qualitative data reveal that students believe they exist.

Although the effects of ‘Engagement with Self‐Reflection’ on the regression model are not statistically significant, these effects are not necessarily insignificant in the world as experienced.^26^ Based on the qualitative data, this rings true as students felt that reflection impacted their self‐efficacy for clinical skills, albeit with no clear consensus on whether the overall effect of reflection on self‐efficacy for clinical skills was positive or negative. Within each theme derived from the interviews and focus groups—‘evaluation of performance’, ‘familiarisation and understanding of skills’, ‘transforms personal mindset’ and ‘connects to emotions’—students had vastly different perceptions of the impacts of reflection on their self‐efficacy. However, Stage 3 of the regression model, with the regression coefficient of Engagement with Self‐Reflection close to zero at −0.023 (vs. 0.362 and 0.399 for the other variables of interest), implied that there is nearly no relationship between engaging with reflection and self‐efficacy for clinical skills. It is possible that the regression model was not discriminating enough to reveal the nuance uncovered during the interviews and focus groups, and hence, these datasets in combination highlight the subtlety of the reflective process—how a variety of effects were subjectively perceived by students but were less tangible then expected by external, objective measures.

Students felt that reflection impacted their self‐efficacy for clinical skills.

These datasets in combination highlight the subtlety of the reflective process.

Perusing the limited available empirical literature on this topic, it appears that this variation in opinions is common. Our study's participants saw reflection as a way to observe their progress in the successful achievement of a skill, a view that was shared by others.^27^ The theme of ‘familiarisation and understanding’ in being a way for reflection to impact clinical skills also echoes sentiments that deeper learning approaches stimulated by reflecting improved self‐efficacy.^28^ However, there are some differences in how our participants perceived the increased awareness of their performance limitations through evaluation. While they felt reflection improved their self‐efficacy by being able to correct areas of weakness, other work has proposed that this realisation would negatively impact self‐efficacy due to additional stress.^28^

The nuanced relationship between engagement with reflection and self‐efficacy may also be explained by the intricacies of the reflection process. Statistically, although the effects of engagement with reflection in predicting self‐efficacy were small and not significant, the ANOVA results suggested that the overall model was still a good fit for our purpose. The other variables added into the model at Stage 2 (Need for Self‐Reflection and Insight), which gave it the largest *R* ^*2*^ change and *p*‐value <0.05, appear to be more potent at predicting self‐efficacy for clinical skills.

Hence, to properly build self‐efficacy for clinical skills, students should understand the purpose of reflecting on their performances and have insight to their actions. As supported by the thematic analysis, reflection may ‘[transform one's] personal mindset’, increasing self‐awareness, which can improve one's self‐efficacy. Having well‐established self‐beliefs also allows students to effectively reflect and learn from their experiences,^29^ and with encouragement and facilitation by trained medical educators, this practice can be enhanced further.^30^

Students should understand the purpose of reflecting on their performances and have insight to their actions.

In summary, from both datasets, the effects of engagement with reflection on one's self‐efficacy for clinical skills are highly variable and are also dependent on factors such as the students' emotional states and cognitive mindsets at the point of reflection. From the themes ‘transforms personal mindset’ and ‘connects to emotions’, internal mental and emotional attributes seem to play a big role in one's self‐efficacy for clinical skills. These qualities are similarly important for academic self‐efficacy amongst medical students.^31^

Internal mental and emotional attributes seem to play a big role in one's self‐efficacy for clinical skills.

Due to the delicate nature of these interweaving factors, students should be nurtured to engage with reflection positively. As illustrated in the theme ‘connects with emotions’, the negative feelings participants reported when reflecting deeply on their mistakes often caused them to overthink. Ruminative thinking is not uncommon in the process of reflection^32^ and can potentially lead to a major blow to one's self‐efficacy for clinical skills. Therefore, reflection, particularly under the guidance of experienced teachers, can ground students by challenging negative patterns and offering solutions,^33^ reinforcing the importance of the medical educator's role in this context.

Students should be nurtured to engage with reflection positively.

Finally, our findings could prove beneficial beyond just the scope of medical students—the strengths of integrating reflective practice into healthcare curricula have previously been described in literature, for example, the use of reflection by nursing students to understand the impact of stressful environments on their emotions and performance and by midwifery students to estimate their self‐efficacy in a key clinical skill.^29^, ^34^ That reflection has the ability to improve one's self‐efficacy is recognised in the core competencies of many health professions; but for students to reap its benefits through all stages of their careers, they should be encouraged to engage with the practice effectively.^35^

## 5 STRENGTHS AND LIMITATIONS

The mixed methods approach allowed the integration of results from two datasets, providing a comprehensive insight into how preclinical students' engagement with reflection affected their self‐efficacy for clinical skills. However, the study's cross‐sectional design only allowed access to the participants' responses at that specific point in time.

A high response rate (94.5%) was representative of the defined study population, although the results may not generalise to other contexts, and there may have been an element of selection bias, with participation reflecting an interest in the benefits of reflection. As the primary researcher who collected the data was a near‐peer, any possible power relationship had a minimal impact on the veracity of the responses, but there was still a chance of recall and reporting bias.

## 6 CONCLUSION

This study has shed some light on the effects of engagement with reflection on self‐efficacy for clinical skills amongst preclinical students. It appears that this relationship varies greatly, whereby engagement with reflection can either improve or reduce one's self‐efficacy. Solely engaging with the process of reflection is insufficient to build self‐efficacy for clinical skills. Hence, reflection should be guided and facilitated by medical educators, while considering factors such as students' perceived needs for the reflective process, their personal mindsets and emotional states.

## AUTHOR CONTRIBUTIONS

**Jade Lene Yong:** Conceptualization; investigation; methodology; software; data curation; validation; formal analysis; visualization; project administration; resources; writing—original draft. **Gwyndaf Roberts:** Conceptualization; methodology; supervision; resources; project administration; formal analysis; validation; visualization.

## CONFLICT OF INTEREST STATEMENT

None.

## ETHICS STATEMENT

Approval for the study was obtained from the Newcastle University Faculty of Medical Sciences Research Ethics Committee (2281/18502/2021). There was no potential harm to participants, anonymity of participants was guaranteed, and informed consent of participants was obtained for publication.

## Supporting information


**Data S1.** Supporting Information

## Data Availability

The data that support the findings of this study are available from the corresponding author upon reasonable request.
