# Supplementary material for: Reflection and self‐efficacy for clinical skills
Source: Clin Teach. 2024 Nov 11;22(1):e13833. doi: 10.1111/tct.13833 (PMC11663727; doi:10.1111/tct.13833)
Supplement: Supplementary file 1 — Data S1. Supporting Information [file TCT-22-e13833-s001.pdf]

Self-Reflection amongst Preclinical Medical Students: Can it Improve Self-Efficacy for Clinical Skills?

Principal Investigator: Yong Jade Lene, Intercalating Medical Student - Master of Medical Education (MMedEd), Newcastle University.

By ticking the box, you are agreeing that you have read and understood the information about the research project as provided in the Information Sheet v2.1, dated 24 March 2022, and that you voluntarily agree to take part in it.

PLEASE TICK THIS BOX: [ ] I agree to take part in this study

Demographics

1. Gender : [ ] Male [ ] Female
2. Age : _____ years
3. Year of Study : [ ] MBBS Year 1 [ ] MBBS Year 2

Self-Reflection and Insight Scale (SRIS)

No.	1 = Strongly disagree; 2 = Disagree; 3 = Neutral; 4 = Agree; 5 = Strongly agree					
	Item					
1	I don’t often think about my thoughts	1	2	3	4	5
2	I am not really interested in analyzing my behaviour	1	2	3	4	5
3	I am usually aware of my thoughts	1	2	3	4	5
4	I am often confused about the way that I really feel about things	1	2	3	4	5
5	It is important for me to evaluate the things that I do	1	2	3	4	5
6	I usually have a very clear idea about why I have behaved in a certain way	1	2	3	4	5
7	I am very interested in examining what I think about	1	2	3	4	5
8	I rarely spend time in self-reflection	1	2	3	4	5
9	I’m often aware that I am having a feeling, but I often don’t quite know what it is	1	2	3	4	5
10	I frequently examine my feelings	1	2	3	4	5
11	My behaviour often puzzles me	1	2	3	4	5
12	It is important to me to try to understand what my feelings mean	1	2	3	4	5
13	I don’t really think about why I behave in the way that I do	1	2	3	4	5
14	Thinking about my thoughts make me more confused	1	2	3	4	5
15	I have a definite need to understand the way my mind works	1	2	3	4	5
16	I frequently take time to reflect on my thoughts	1	2	3	4	5
17	Often, I find it difficult to make sense of the way I feel about things	1	2	3	4	5
18	It is important to me to be able to understand how my thoughts arise	1	2	3	4	5
19	I often think about the way I feel about things	1	2	3	4	5
20	I usually know why I feel the way I do	1	2	3	4	5

Self-Reflection amongst Preclinical Medical Students: Can it Improve Self-Efficacy for Clinical Skills?

Learning Self-Efficacy Scale (L-SES) for Clinical Skills

No.	**1 = Strongly disagree; 2 = Disagree; 3 = Neutral; 4 = Agree; 5 = Strongly agree**					
	Item					
1	I can recall how to perform clinical skills I have learnt.	1	2	3	4	5
2	I understand the content of clinical skills I have learnt and can demonstrate it to others.	1	2	3	4	5
3	I can verbally explain the purpose and principle of operating clinical skills I have learnt.	1	2	3	4	5
4	I can verbally explain the sequence and interrelationship between each step of clinical skills I have learnt.	1	2	3	4	5
5	I think I spend more time on learning clinical skills than on other aspects of the MBBS course.	1	2	3	4	5
6	I think I gain more in learning clinical skills than in other aspects of the MBBS course.	1	2	3	4	5
7	I tend to pay more attention to information related to learning clinical skills.	1	2	3	4	5
8	I tend to actively look for information related to learning clinical skills.	1	2	3	4	5
9	I can precisely imitate the instructor's steps and actions of clinical skills I have learnt.	1	2	3	4	5
10	I can smoothly complete the operation steps of clinical skills I have learnt.	1	2	3	4	5
11	I try to monitor my clinical skills for improvements.	1	2	3	4	5
12	I try to monitor my clinical skill operations and make proper adjustments as needed.	1	2	3	4	5

Thank you for your participation!
Please hand in your completed questionnaire to the tutor in-charge of your clinical skills session.

As the topics of self-reflection and self-efficacy are personal and introspective, the researcher understands that this survey may prompt students to consider their academic development in medical school thus far. Hence, the researcher would like to take the opportunity to remind students that formal support is available via the university's academic mentors and tutors, should you wish to speak to anyone regarding such concerns.

Here, the researcher would also like to reassure you that uncertainties are not uncommon in our medical school journeys. From one medical student to another, personal and academic growth truly happens over time, whether we realize it or not. All the best in your studies!

QR Code for Participant Information Sheet
